# Comparing modes of delivery of a combination of ion channel inhibitors for limiting secondary degeneration following partial optic nerve transection

**DOI:** 10.1038/s41598-019-51886-3

**Published:** 2019-10-25

**Authors:** Lillian M. Toomey, Carole A. Bartlett, Nikolas Gavriel, Terence McGonigle, Maimuna Majimbi, Gopana Gopalasingam, Jennifer Rodger, Melinda Fitzgerald

**Affiliations:** 1 Experimental and Regenerative Neurosciences, School of Biological Sciences, The University of Western Australia, 35 Stirling Hwy, Perth, 6009 Western Australia Australia; 2 Curtin Health Innovation Research Institute, Curtin University, Sarich Neuroscience Research Institute Building, 8 Verdun St, Nedlands, 6009 Western Australia Australia; 3 Perron Institute for Neurological and Translational Science, Sarich Neuroscience Research Institute Building, 8 Verdun St, Nedlands, 6009 Western Australia Australia

**Keywords:** Preclinical research, Ion channels in the nervous system

## Abstract

Injury to the central nervous system is exacerbated by secondary degeneration. Previous research has shown that a combination of orally and locally administered ion channel inhibitors following partial optic nerve injury protects the myelin sheath and preserves function in the ventral optic nerve, vulnerable to secondary degeneration. However, local administration is often not clinically appropriate. This study aimed to compare the efficacy of systemic and local delivery of the ion channel inhibitor combination of lomerizine, brilliant blue G (BBG) and YM872, which inhibits voltage-gated calcium channels, P2X_7_ receptors and Ca^2+^ permeable α-amino-3-hydroxy-5-methyl-4-isoxazolepropionic acid (AMPA) receptors respectively. Following a partial optic nerve transection, adult female PVG rats were treated with BBG and YM872 delivered *via* osmotic mini pump directly to the injury site, or *via* intraperitoneal injection, both alongside oral administration of lomerizine. Myelin structure was preserved with both delivery modes of the ion channel inhibitor combination. However, there was no effect of treatment on inflammation, either peripherally or at the injury site, or on the density of oligodendroglial cells. Taken together, the data indicate that even at lower concentrations, the combinatorial treatment may be preserving myelin structure, and that systemic and local delivery are comparable at improving outcomes following neurotrauma.

## Introduction

Damage to the CNS, known as neurotrauma^1^, occurs in the context of traumatic brain and spinal cord injuries as well as optic neuropathy, leading to substantial loss of function for which there is currently no effective therapy. Cells adjacent to the injury site are vulnerable to secondary degeneration, a self-propagating cascade of reactive pathways characterised by glutamate- and ATP-induced excitotoxicity, intracellular Ca^2+^ overload, oxidative stress, microglial and macrophage activation and infiltration, dysmyelination and cellular apoptosis^2–4^. Secondary degeneration has been shown to spread to initially undamaged tissue and is associated with chronic functional loss^5^. Therefore, therapeutic strategies to minimise damage following neurotrauma need to limit this secondary degeneration of tissue adjacent to the primary injury.

In order to develop effective treatments for limiting secondary degeneration, therapeutics need to be trialled in appropriate animal models of injury, and partial optic nerve transection has been utilised for this purpose. In this model, the right dorsal optic nerve of rats is partially transected leaving the ventral nerve vulnerable to secondary degeneration, and allowing for spatial segregation between the primary and secondary injury sites^6,7^. This particular model has been utilised in pre-clinical studies whereby therapeutics for secondary degeneration are directly delivered to the injury site *via* an implanted osmotic mini-pump drug delivery system^5,8–10^.

Following milder injuries to the central nervous system there is contradictory literature as to what extent the blood-brain barrier (BBB) is breached^11,12^ or whether the BBB is compromised at all^13^. Nevertheless, efficacy of treatments for neurotrauma are frequently assessed in pre-clinical models that employ delivery systems to administer agents directly to an injury site, and the ability of an agent to access the brain from the periphery is not always considered. The partial optic nerve transection model results in disruption of the BBB at the optic nerve injury site for at least 3 days after injury^14^. As such, at 3 days after injury the model allows for direct pre-clinical assessment of therapeutics for neurotrauma and proof of principle investigations into treatments for secondary degeneration specifically, in which comparisons can be made between local and systemic delivery of agents where the blood brain barrier is known to be breached.

Using the partial optic nerve transection model, we have previously investigated a combinatorial treatment employing the ion channel inhibitors lomerizine, Brilliant Blue G (BBG) and YM872. This treatment is designed to limit intracellular Ca^2+^ influx through voltage-gated calcium channels (VGCC)^15^, P2X_7_ receptors^16^ and Ca^2+^ permeable α-amino-3-hydroxy-5-methyl-4-isoxazolepropionic acid (AMPA) receptors^17^ respectively. When YM872 and BBG were locally delivered directly to the injury site *via* osmotic mini-pumps for 3 days following injury, alongside oral administration of lomerizine, we found that this treatment preserved function and partially protected the structure of the node of Ranvier^10^. All three ion channel inhibitors can cross the BBB^18–21^, making it clinically suitable for systemic delivery. Therefore, this study aimed to compare the efficacy of local and systemic delivery of this combinatorial treatment for limiting secondary degeneration following a partial optic nerve transection, assessing inflammatory cell and oligodendroglial populations as well as structure of the node of Ranvier.

## Results

### Effects of ion channel inhibitor combinations on microglia and macrophages

**Figure 1:**
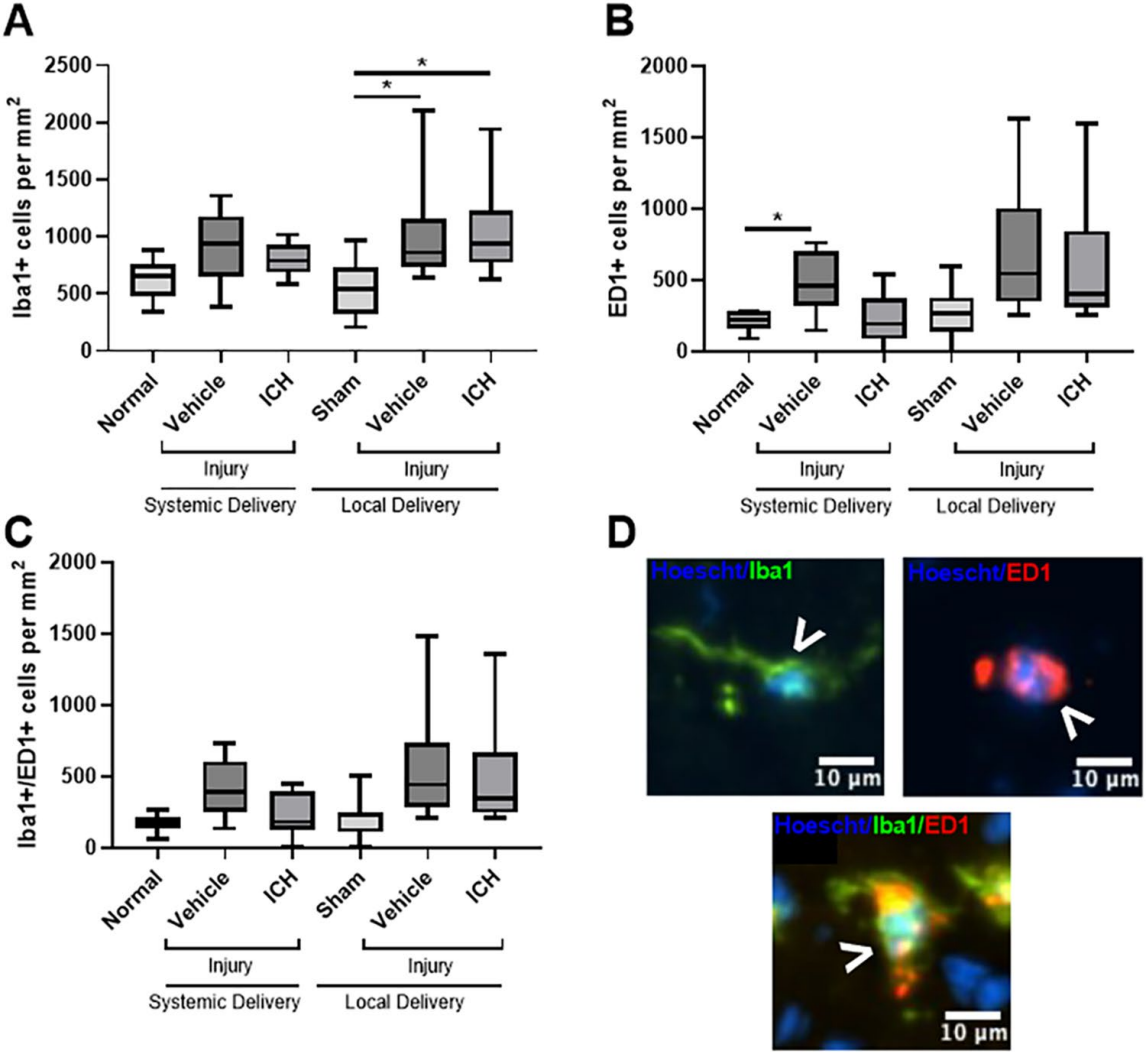
Effects of ion channel inhibitor combinations on densities of Iba1+ and ED1+ cells. Densities of Iba1+ (**A**), ED1+ (**B**) and Iba1+/ED1+ (**C**) cells in the ventral optic nerve from uninjured normal; sham injured, local vehicle treated animals; injured, systemic and local vehicle treated animals; and systemic and local ion channel inhibitor treated animals 3 days after partial optic nerve transection. N = 8–10 rats per group; graphs display min to max values, with the central line representing the median data point. Significant differences are indicated by *p ≤ 0.05. (**D**) Representative images of Iba1+, ED1+ and Iba1+/ED1+ cells, indicated with arrow heads; scale bars = 10 µm.

To examine the effect of the ion channel inhibitor combination on local inflammation, changes in the cell densities of Iba1+ resident ramified microglia, ED1+ infiltrating phagocytic microglia/macrophages and Iba1+/ED1+ infiltrating activated microglial cells^22–24^ were quantified in the ventral optic nerve following a partial optic nerve transection relative to controls (Fig. 1D). Injury caused a significant increase in the number of Iba1+ resident ramified microglia in the presence of the osmotic mini-pump locally delivering vehicle (Fig. 1A; F = 3.624, df = 5, p = 0.026); this increase was not observed when vehicle treatment was delivered systemically *via* intraperitoneal injections (p = 0.464). The number of Iba1+ resident ramified microglia in the locally delivered ion channel inhibitor treatment group remained significantly increased compared to the uninjured, sham group (p = 0.026). Neither delivery mode of ion channel inhibitor treatment resulted in significant decreases in numbers of resident ramified microglia relative to their respective injured vehicle treated groups (systemic p = 0.978, local p = 1.000). There were no significant differences observed in the number of Iba1+ resident ramified microglia between the two uninjured control groups (p = 0.992) or between the two injured vehicle administration groups (p = 0.970).

Injury with systemic vehicle administration resulted in a significant increase in the number of ED1+ infiltrating phagocytic microglia/macrophage cells relative to uninjured controls (Fig. 1B; F = 4.357, df = 5, p = 0.038). However, injury combined with an osmotic mini-pump delivering vehicle did not show a significant increase in the level of ED1+ cells (p = 0.115), perhaps due to increased variability in inflammation caused by the delivery catheter at the injury site (Fig. 1B). Neither systemic nor local treatment with the ion channel inhibitors significantly reduced the numbers of ED1+ infiltrating phagocytic microglia/macrophage cells compared to the vehicle treatment groups (systemic p = 0.111, local p = 0.997). There were no significant differences observed in the number of ED1+ infiltrating phagocytic microglia/macrophage cells between the two uninjured control groups (p = 0.968) or between the two injured vehicle administration groups (p = 0.789).

Finally, injury caused a trend towards an increase in the numbers of Iba1+/ED1+ infiltrating activated microglial cells in the systemically delivered vehicle group (F = 3.615, df = 5, p = 0.053), but not in the injured group with osmotic mini-pumps delivering vehicle (p = 0.144). Neither delivery mode resulted in significant reductions in Iba1+/ED1+ infiltrating activated microglial cells with the ion channel inhibitors compared to their respective vehicle groups (systemic p = 0.279, local p = 0.998). There were no significant differences observed in the number of Iba1+/ED1+ infiltrating activated microglial cells between the two uninjured control groups (p = 0.990) or between the two injured vehicle administration groups (p = 0.900).

### Effects of ion channel inhibitor combinations on plasma cytokine and chemokine concentrations

**Figure 2:**
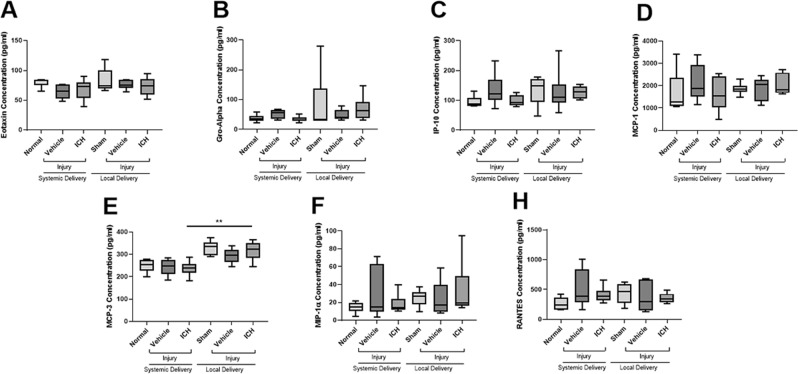
Effects of injury and ion channel inhibitor combinations on the plasma concentration of cytokine and chemokine analytes. Concentration of MIP-2 (**A**), IP-10 (**B**), RANTES (**C**), MIP-1α (**D**), MCP-3 (**E**), MCP-1 (**F**), and Eotaxin (**G**) in the plasma of uninjured normal; sham injured, local vehicle treated animals; injured, systemic and local vehicle treated animals; and systemic and local ion channel inhibitor treated animals 3 days after partial optic nerve transection. Graphs display min to max values, with the central line representing the median data point; N = 5–6 rats per group. Significant differences indicated by *p ≤ 0.05 and ***p ≤ 0.001.

To determine whether the ion channel inhibitor combinations were having an effect on inflammation in the periphery, the effects of the ion channel inhibitor combination on the expression of 22 cytokines and chemokines were assessed in plasma samples. 15 of the analytes were below the detection limit of the assay and were excluded from analysis: i.e. IL-1α (interleukin 1 alpha), G-CSF (granulocyte-colony stimulating factor), IL-10 (interleukin 10), IL-1β (interleukin 1 beta), IL-6 (interleukin 6), TNFα (tumour necrosis factor alpha), IL-4 (interleukin 4), GM-CSF (granulocyte-macrophage colony-stimulating factor), IFNγ (interferon gamma), IL-2 (interleukin 2), IL-5 (interleukin 5), IL-13 (interleukin 13), IL-12p70 (interleukin 12p70), and MIP-2 (macrophage inflammatory protein 2); data not shown. Of those analysed, significant differences between groups were found for only one of the analytes, MCP-3 (monocyte-chemotactic protein 3; Fig. 2). While injury did not result in a significant effect on MCP-3 concentration (F = 8.084, df = 5) for either systemic (p = 0.999) or local vehicle delivery (p = 0.467), the systemically delivered ion channel inhibitor combination did result in a significantly decreased concentration of MCP-3 compared to when the ion channel inhibitor combination was locally delivered (p = 0.005). Neither delivery mode of the ion channel inhibitor combination affected the concentration of MCP-3 relative to their respective vehicle controls (systemic p = 1.000, local p = 0.846). There was a significant difference observed in the concentration of MCP-3 between the two uninjured control groups (p = 0.005), but there was no difference between groups for the two injured vehicle administration groups (p = 0.151). This result likely reflects the increased inflammation caused by the catheter attached to the osmotic mini-pump. There were no significant differences detected in the plasma concentrations of Eotaxin (F = 1.478, df = 5), Gro-α (growth-regulated oncogene alpha; F = 0.917, df = 5), IP-10 (interferon gamma-induced protein 10; F = 1.023, df = 5), MCP-1 (monocyte chemoattractant protein-1; F = 0.507, df = 5), MIP-1α (macrophage inflammatory protein 1 alpha; F = 0.722, df = 5), or RANTES (regulated on activation normal T cell expressed and secreted; F = 1.089, df = 5).

### Effects of ion channel inhibitor combinations on oligodendroglia

**Figure 3:**
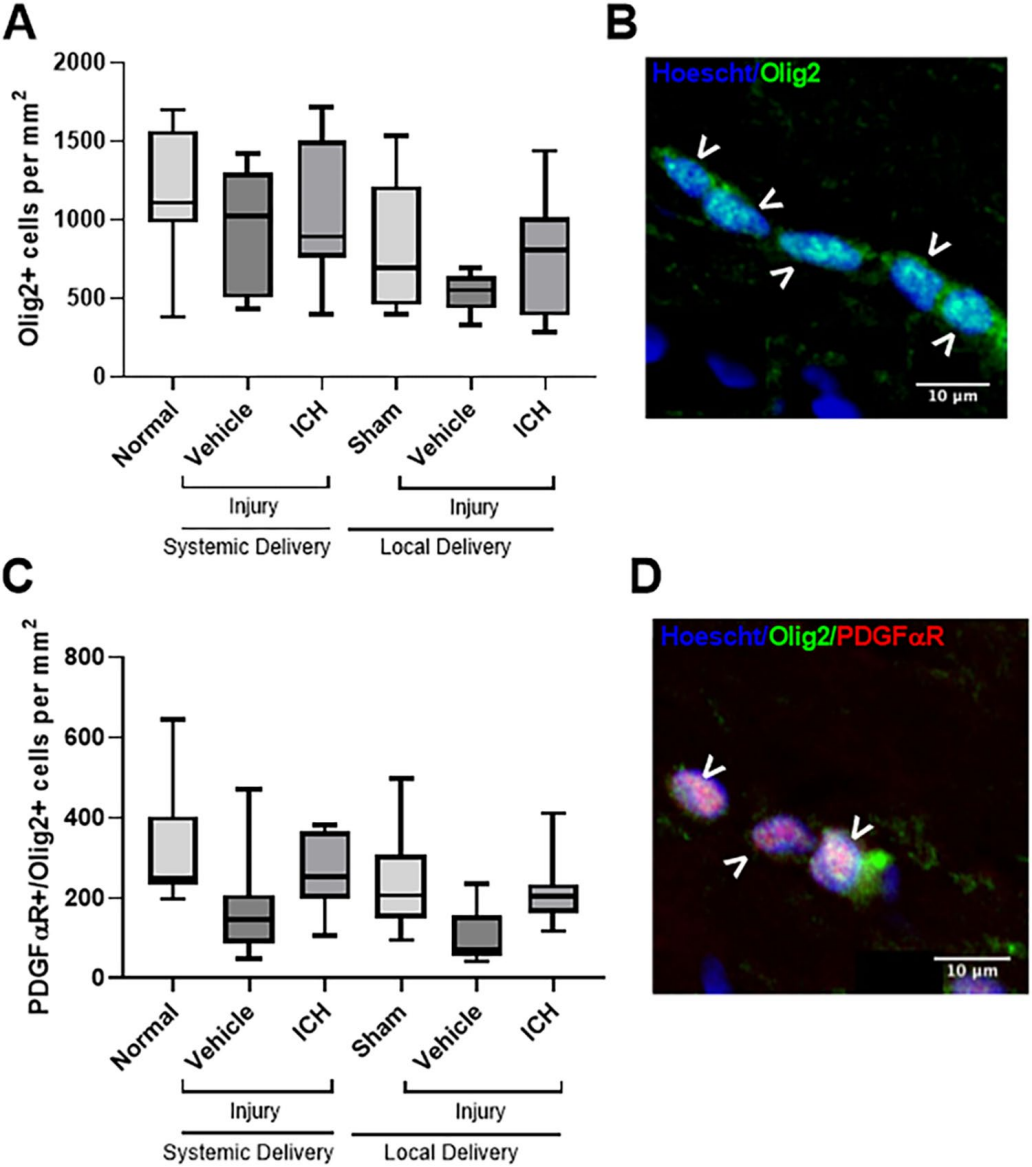
Effects of ion channel inhibitor combinations on oligodendroglial cells. Densities of Olig2+ oligodendroglial cells (**A**) and PDGFαR+/Olig2+ OPCs (**C**) in the ventral optic nerve from uninjured normal; sham injured, local vehicle treated animals; injured, systemic and local vehicle treated animals; and systemic and local ion channel inhibitor treated animals 3 days after partial optic nerve transection. N = 8–10 rats per group. Graphs display min to max values, with the central line representing the median data point. Representative image of Olig2+ cells (**B**) and OPCs (**D**), indicated with arrow heads; scale bars = 10 µm.

Oligodendroglia are particularly vulnerable to damage following neurotrauma^25^, and to examine the effect of the ion channel inhibitor combination on oligodendroglial cell numbers, the densities of Olig2+ cells were quantified (Fig. 3B). There were no effects of injury with either mode of vehicle administration, compared to the relevant uninjured control group, on the densities of Olig2+ oligodendroglial cells (Fig. 3A; F = 3.308, df = 5, systemic p = 0.816, local p = 0.442). Treatment with the ion channel inhibitor combination administered *via* either delivery mode also did not change the densities of oligodendroglial cells (systemic p = 0.980, local p = 0.674). There were no significant differences observed in the density of Olig2+ oligodendroglial cells between the two uninjured control groups (p = 0.612) or between the two injured vehicle administration groups (p = 0.143).

When analysing oligodendrocyte precursor cells (OPCs) specifically, by detection of Olig2 together with PDGFαR (Fig. 3D), there were also no effects of injury with either mode of vehicle administration when compared to their uninjured controls (Fig. 3C; F = 4.344, df = 5, systemic p = 0.063, local p = 0.126). Treatment with the ion channel inhibitor combination delivered by either mode also did not change the number of OPCs relative to relevant vehicle administered controls (systemic p = 0.527, local p = 0.222). There were no significant differences observed between the two delivery modes for the ion channel inhibitor treatments (p = 0.958). There were also no significant differences observed in the density of OPCs between the two uninjured control groups (p = 0.597) or between the two injured vehicle administration groups (p = 0.751).

### Effects of ion channel inhibitor combinations on node/paranode complexes

**Figure 4:**
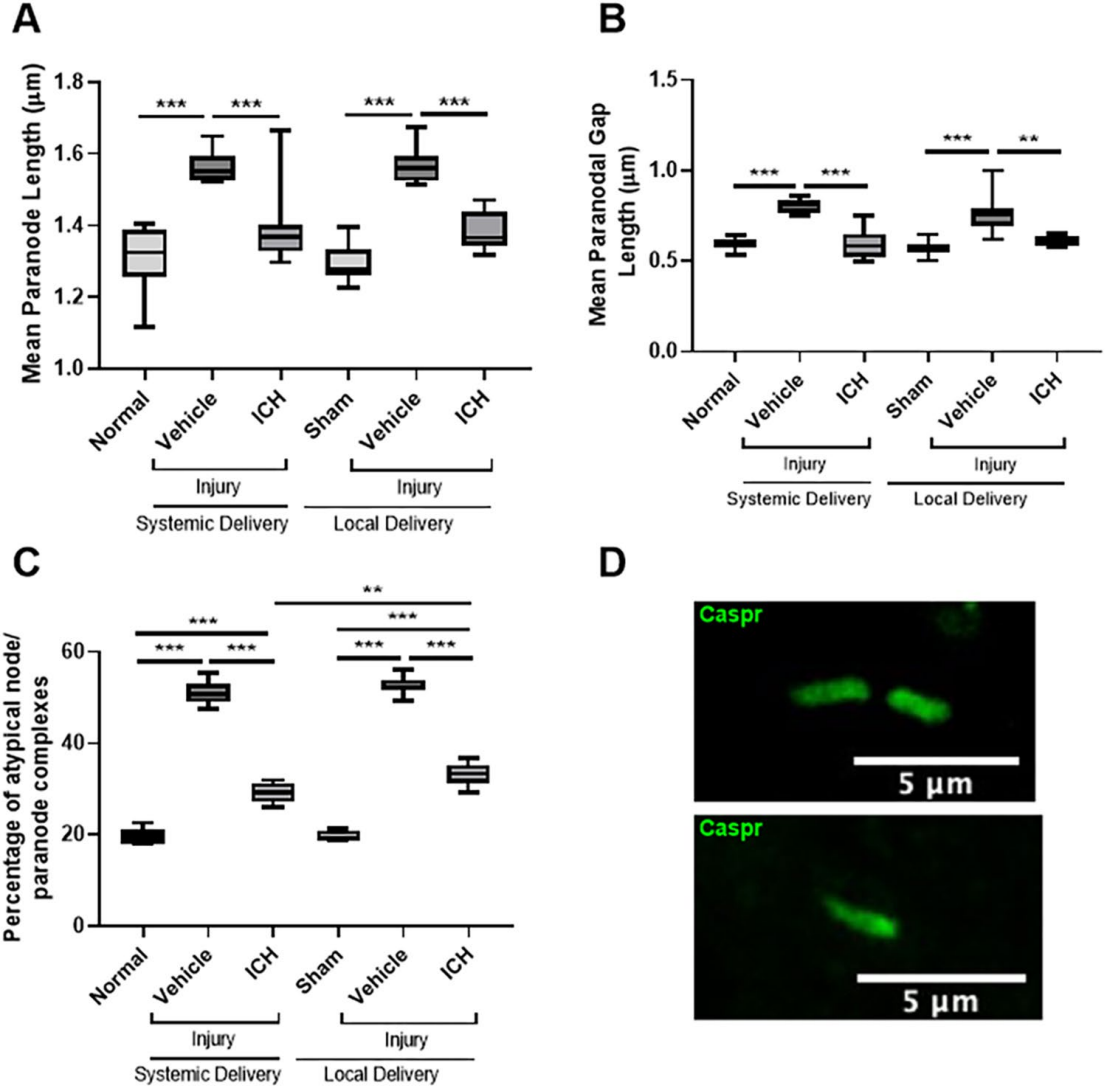
Effect of ion channel inhibitor combinations on node/paranode complexes. Paranode length (**A**), paranodal gap length (**B**), and percentage of atypical node/paranode complexes (**C**) from at least thirty nodal complexes per animal in the ventral optic nerve from uninjured normal; sham injured, local vehicle treated animals; injured, systemic and local vehicle treated animals; and systemic and local ion channel inhibitor treated animals 3 days after partial optic nerve transection. Graphs display min to max values, with the central line representing the median data point; N = 8–10 rats per group. Significant differences indicated by *p ≤ 0.05, **p ≤ 0.01, and ***p ≤ 0.001. (**D**) Representative image of two Caspr +areas flanking a β-III tubulin+ area or a typical nodal complex; scale bar = 2 µm. (**E**) Representative image of one Caspr+ area flanking a β-III tubulin+ area denoting an atypical nodal complex or a heminode; scale bars = 5 µm.

To determine the effects of the ion channel inhibitor combination on structure of the node of Ranvier following injury, the length of the paranode and the paranodal gap, indicative of the length of the Node of Ranvier, were measured, along with the proportion of atypical nodal complexes (Fig. 4). The paranode length increased with injury when vehicle was delivered either systemically (Fig. 4A; F = 25.089, df = 5, p ≤ 0.001) or locally (p ≤ 0.001). Both modes of delivery of the ion channel inhibitors resulted in significantly reduced paranode length compared to the relevant vehicle treatment groups (both p ≤ 0.001), to paranode lengths not significantly different from their relevant uninjured controls (systemic p = 0.197, local p = 0.088). There were no significant differences observed in the length of the paranode between the two uninjured control groups (p = 0.996) or between the two injured vehicle administration groups (p = 1.000).

Injury also significantly increased the length of the paranodal gap following both systemic (Fig. 4B; F = 22.898, df = 5, p ≤ 0.001) and local (p ≤ 0.001) vehicle administration. Both modes of delivery of the ion channel inhibitors significantly reduced the paranodal gap length compared to the outcome following relevant vehicle administration (p ≤ 0.001) to paranodal gap lengths not significantly different from their relevant uninjured controls (systemic p = 1.000, local p = 0.683). There were no significant differences observed in the length of the paranodal gap between the two uninjured control groups (p = 0.966) or between the two injured vehicle administration groups (p = 0.589).

Finally, there was a significant increase in the percentage of atypical node/paranode complexes with injury following both systemic and local vehicle administration compared to the relevant uninjured controls (Fig. 4C; F = 445.292, df = 5, both p ≤ 0.001). Both modes of delivery of the ion channel inhibitors significantly reduced the percentage of atypical complexes compared to relevant vehicle treatment (both p ≤ 0.001), however both remained significantly higher than the relevant uninjured control group (both p ≤ 0.001). Interestingly, treatment with the systemically administered ion channel inhibitors significantly reduced the percentage of atypical node/paranode complexes compared to local administration (p = 0.002). There were no significant differences observed in the percentage of atypical complexes between the two uninjured control groups (p = 1.000) or between the two injured vehicle administration groups (p = 0.583).

## Discussion

Following optic nerve injury, systemic delivery of a combination of ion channel inhibitors was just as effective at preserving the structure of the node of Ranvier in optic nerve vulnerable to secondary degeneration, as when two of the agents were administered directly to the injury site. Preservation of node of Ranvier structure has been demonstrated to be associated with preservation of function of the optic nerve, in the form of preservation of the number of visual responses in the optokinetic nystagmus test, including following local administration of this combination of ion channel inhibitors^10^. Although a local inflammatory response was observed with injury alone, in line with our previous work that has demonstrated increased microglia and macrophages at both the primary injury site along the optic nerve and remotely to the visual centres in the brain^26,27^, there was no effect of the inhibitor combination on inflammation with either mode of delivery, either at the injury site or peripherally in the circulatory system. The lack of effect of the inhibitor combination is in line with studies assessing a similar combination of inhibitors in this model, showing that the ion channel inhibitor combination YM872, oxATP and lomerizine, when locally delivered for three days following injury as performed in the current study, has no significant effect on the number of Iba1+ and ED1+ cells at the primary injury site or in the ventral optic nerve vulnerable to secondary degeneration^9^ Furthermore, the current study extends outcomes to demonstrate no discernible changes in circulating chemokines either with injury or ion channel inhibitor treatment. There were also no changes observed in the numbers of oligodendroglial cells either with injury or with treatment. Overall, this indicates that the ion channel inhibitor combination is not working *via* ameliorating the immune response, but perhaps acting on the myelin sheath directly.

Myelin structural changes are a key feature of neurotrauma, with chronic dysmyelination observed following injury^28,29^. Myelin degeneration has previously been associated with P2X_7_ receptor activation^30^, and P2X_7_ receptor antagonists have been shown to protect white matter following injury^31,32^. Ca^2+^ permeable AMPA receptors^33^ are present on the myelin sheath, and myelin degeneration further exposes sub-myelin VGCCs^34^. Oligodendroglia themselves have a higher concentration of AMPA and P2X_7_ receptors than other neuroglia^35,36^. Furthermore, oligodendroglia do not appear to possess the ability for receptor-mediated desensitisation of AMPA receptors during periods of overstimulation, thus increasing their risk of excitotoxic insult^37^. Once the cell depolarises upon entry of Ca^2+^, this activates VGCCs which results in further Ca^2+^ influx^38^. A similar ion channel inhibitor combination, comprised of lomerizine, YM872 and the alternative P2X_7_ receptor antagonist oxATP, has been assessed *in vitro* and was found to successfully limit excess Ca^2+^ entry^39^. Taken together with the outcomes of this study, the data suggest that control of Ca^2+^ influx into cells is important for the preservation of the structure of the node of Ranvier. While the ion channel inhibitor combination of lomerizine, YM872 and oxATP has been shown to preserve axons both acutely^9^ and chronically^5^, densities of retinal ganglion cell somata were intermediate and not significantly different from densities in either normal or injured animals^5^. The data suggest that preservation of the myelin nodal complex does not necessarily guarantee retinal ganglion cell survival.

Excess intracellular Ca^2+^ contributes to mitochondrial dysfunction and subsequent oxidative damage, including lipid peroxidation^40^. Oxidative damage does not always cause oligodendroglial death^28^, but does cause oligodendroglial dysfunction and dysmyelination^41^. Being a highly lipid-rich structure^42^, myelin is extremely vulnerable to lipid peroxidation as a result of oxidative stress^43^. By three days following a partial optic nerve transection, there is a significant increase in the levels of 4-hydroxynonenal, a marker of lipid peroxidation, colocalising with myelin basic protein^44^, suggesting lipid peroxidation of the myelin sheath is a hallmark of white matter injury following neurotrauma. Lipid peroxidation of the myelin sheath also results in the aggregation and instability of myelin basic protein, resulting in further dysmyelination^45^. Therefore, the observed preservation of node of Ranvier structure with treatment with the ion channel inhibitor combination may be due to reduced lipid peroxidation, which has also been observed when using a similar combination of inhibitors^9^.

The concentration of the ion channel inhibitor combination that would have reached the injury site when the combination was systemically delivered would likely be below that when the combination was delivered directly to the nerve^46^. Based on the blood volume of a rat, and if 100% of the agent was absorbed into the bloodstream from the intraperitoneal injection, the blood concentration would be 823.31μM BBG and 943.41μM YM872. However, while data on blood concentration of the agents employed following intraperitoneal injection are not available, a range of studies indicate that the average concentration of agents in blood is only 0.5% of that intraperitoneally injected^47–49^. Therefore, the amount that would have reached the injury site is likely to have been considerably less than the 540 μM BBG and 240 μM YM872 delivered using injury site administration. Overall, this suggests that the ion channel inhibitor combination at the injury site may be at least as effective at lower concentrations.

Local drug delivery has limited clinical utility due to the difficulties associated with surgical implantation of delivery devices, risk of infection and the limited time for which devices can remain *in situ*^50^. Systemic delivery of pharmacotherapeutics is generally preferred due to fewer delivery associated side effects^46^. Nevertheless, systemic delivery is often hindered by the presence of the BBB, which limits entry of many pharmacotherapeutics into the CNS^51^.

This study demonstrated the efficacy of a clinically relevant systemically delivered ion channel inhibitor combination for limiting secondary degeneration following a partial optic nerve transection, when the BBB is known to be breached^14^. In most rodent models of mild traumatic brain injury, BBB dysfunction typically resolves within minutes to days^52–55^, however some studies have found no BBB dysfunction following injury^13^. In human studies of mild traumatic brain injury, changes in BBB integrity have been found to be heterogeneous^11,56^, with not all patients showing increased markers of BBB dysfunction following injury^57^, whilst other patients displaying BBB dysfunction years following injury^58^. Therefore, pharmacotherapies designed to treat neurotrauma need to possess the ability to cross a closed BBB to reach the site of damage. All three of the ion channel inhibitors in the combination assessed in the current study are able to cross the BBB^18–21,59^. Therefore, this BBB permeable treatment may also be suitable for milder traumatic brain injuries, such as concussion, where the BBB is likely to only be transiently open^55,56^.

## Methods

### Animals and study design

Adult, female PVG rats were acquired from the Animal Resource Centre in Murdoch, Western Australia. Animals were housed with *ad libitum* access to water and food under a 12-hour light/dark cycle. All procedures were approved by the University of Western Australia Animal Ethics Committee (Approval Number RA3/100/1485) and were in accordance with the National Health and Medical Research Council (NHMRC) of Australia Code of Practice for use of Animals for Scientific Purposes. Two cohorts of animals were utilised for this study. The first cohort had fifty-six rats that were divided into six experimental groups; a normal group (n = 8), an injured, systemically delivered vehicle group (n = 10), an injured, systemically delivered ion channel inhibitor combination treatment group (n = 10), an injured, locally delivered vehicle group (n = 10), an injured, ion channel inhibitor combination treated group (n = 10) and a sham injured, locally delivered vehicle group (n = 8). The normal group served as an uninjured control for systemic delivery and the sham group served as an uninjured control for local delivery; n’s are noted in the Figure legends. The second cohort had 48 rats (n = 8/group), with the same six experimental groups as cohort 1. The tissue from cohort 1 was processed for immunohistochemical analysis and bloods collected from cohort 2 were processed for a multiplex cytokine analysis.

### Surgical procedures

Partial optic nerve transection (day 0) was performed as previously described^8,60^, under intraperitoneal Ketamine (Ketamil, 50 mg/kg, Troy Laboratories) and Xylazine (Ilium Xylazil, 10 mg/kg, Troy Laboratories) anaesthesia. In summary, the skin overlying the skull behind the right eye was incised and retracted forwards to allow access to the orbit. About 1 mm behind the right eye, the optic nerve parenchyma was exposed by making a longitudinal cut in the sheath using fine iridectomy scissors. The dorsal aspect of the optic nerve was partially lesioned using a diamond radial keratotomy knife (Geuder) to a depth of approximately 200 μm. Sham injury included all procedures except the cut in the sheath and the partial optic nerve lesion. Implantation of Alzet osmotic mini-pumps was performed as described^5^. Subcutaneous injections of analgesia (2.8 mg/kg carprofen, Norbrook) and 1 mL sterile phosphate buffered saline (PBS) were administered to the animals directly following surgery.

### Treatments

Lomerizine (30 mg/kg, LKT Labs©) was orally administered in butter vehicle twice daily 8 hours apart for the duration of the experiment as previously described^8^. For local delivery, BBG (540 μM) and YM872 (240 μM) in PBS vehicle were delivered *via* osmotic mini-pump at a rate of 0.5 μL/h. The concentrations employed for local delivery were consistent with our previous studies where efficacy was demonstrated^5,10^. The sham injured and partial optic nerve transection injured, locally delivered vehicle treated experimental groups both received PBS *via* osmotic mini-pump and orally administered butter vehicle. For systemic delivery, BBG (45 mg/kg, Sigma-Aldrich) and YM872 (20 mg/kg, LKT Laboratories) in PBS were administered intraperitoneally 2 hours post-surgery on day 1, and in the afternoon of day 3. The dosages of systemically administered BBG and YM872 were based on previous studies^17,61,62^. The partial optic nerve transection injured, systemically delivered vehicle treated experimental group received an equivalent volume intraperitoneal injection of PBS and butter orally, administered as described for the inhibitor treated group. The normal group did not receive any treatments.

### Tissue processing

Three days after partial optic nerve transection, rats were euthanised with pentobarbitone sodium (160 mg/kg, Delvet). Rats in cohort 1 were transcardially perfused with 0.9% saline, followed by 4% paraformaldehyde (PFA; Sigma-Aldrich) in 0.1 M phosphate buffer. Optic nerves were dissected and fixed overnight in 4% PFA and subsequently transferred into 15% sucrose (Chem Supply), 0.1% sodium azide (Sigma-Aldrich) in PBS pH 7.2 for storage. Tissue was then cryosectioned longitudinally to a thickness of 14 µm and collected onto Superfrost Plus glass microscope slides. In cohort 2, blood samples were taken *via* cardiac puncture from the rats following euthanasia. Blood samples were collected into heparinised tubes and spun at 3000 rpm, 4 °C for 10 minutes. Plasma was separated and stored at −80 °C until subsequent multiplex assay analysis.

### Immunohistochemistry

Immunohistochemical analysis was conducted on tissue obtained from cohort 1 in accordance with established procedures^26^. Primary antibodies were used that recognised: microglial activation markers Iba1 (1:500; Abcam, goat Ab5076) and ED1 (1:500; Merck Millipore, mouse MAB1435); oligodendroglial indicators oligodendrocyte transcription factor 2 (Olig2; 1:500; R&D Systems, goat AF2418) and platelet-derived growth factor alpha receptor (PDGFαR; 1:500; Abcam Ab96806); and for paranode and node of Ranvier structures, Caspr (1:500; Abcam, rabbit Ab34151). Antibodies were diluted in PBS containing 0.2% Triton™ X-100 and 5% normal donkey serum. Secondary antibodies were Alexa Flour 488 or 555 (1:400; Thermo Fisher Scientific™), with Hoechst 3342 (1:1000; Thermo Fisher Scientific™), diluted in PBS containing 0.2% Triton™ X-100. Finally, the sections were mounted and cover slipped using Fluoromount-G (Thermo Fisher Scientific).

### Imaging and analysis

Imaging was performed as previously described^10^. The ventral optic nerve directly below the site of injury was visualised using either a Nikon Ni-E confocal fluorescence microscope (Nikon Corporation) or a Nikon Eclipse Ti inverted microscope, with one field of view from one section per animal imaged for each outcome measure. A series of 13 optical images were taken at 0.5μm increments along the z-axis, and deconvoluted using Nikon Elements AT software. Imaging for each outcome measure was performed in a single session with consistent capture settings. All image analysis was performed on Fiji image processing software (NIH) by a single investigator blinded to tissue identity.

Total numbers of Iba1+ resident ramified microglia, ED1+ activated phagocytic microglia/macrophages, Iba1+/ED1+ infiltrating activated microglia^22–24^, Olig2+ oligodendroglia and Olig2+/PDGFαR+ oligodendrocyte precursor cells (OPCs) were counted within a region of interest in a 20x image of the ventral nerve directly beneath the primary injury site and expressed as the mean number of cells/mm^2^. For node/paranode analyses, a single 60x z-stack image per animal was divided into a 3 × 3 grid and complexes with clearly defined Caspr immunostaining in a single randomly selected grid square were analysed. At least thirty complexes per animal were assessed for the length of the paranodal gap, defined as the distance between two Caspr+ areas; paranode length, as defined by the length of Caspr+ areas: the percentages of atypical nodal complexes were determined in the entire 60x image, all as previously described^28^.

### Multiplex assay

The concentration of 22 cytokine and chemokines (IL-1α, G-CSF, IL-10, IL-17A, IL-1β, IL-6, TNFα, IL-4, GM-CSF, IFNγ, IL-2, IL-5, IL-13, IL-12p70, Eotaxin, GROα, IP-10, MCP-1, MCP-3, MIP-1α, MIP-2, and RANTES) were assessed in the plasma samples collected from cohort 2, using a Cytokine and Chemokine 22-Plex Rat ProcartaPlex^TM^ Panel (Invitrogen) according to manufacturer’s instructions. 6 plasma samples per group were chosen using random sampling from the cohort of n = 8 and assessed in duplicate, alongside seven antigen standards in duplicate and two blank wells. The plate was then run on a BioPlex^®^ 200 system and the amount of analyte plotted against the standard curve.

### Statistics

Results were analysed using IBM SPSS software. Single one-way ANOVAs encompassing all six treatment groups were performed for each outcome measure and F and degrees of freedom (dF) outcomes described at first mention in the Results section. Normality was assumed, Levene’s test was used to assess homogeneity of variances for each data set (α = 0.05). If Levene’s test showed equal variance, the Tukey post-hoc was used; for unequal variance, the Games-Howell post-hoc was applied; both used p ≤ 0.05 to indicate statistical significance and these p values are provided in the results text. Statistical significances shown on graphs are hypothesis driven and may not display all significant differences obtained. Specifically, only significant differences within systemic and local delivery groups are presented on graphs, as well as differences between the local and systemic ion channel inhibitor treatment groups. No outliers were removed for any of the outcome measures; missing data points in the multiplex analyses were due to values outside of the detection limit of the assay.

## Data Availability

The datasets generated during and/or analysed during the current study are available from the corresponding author on reasonable request.
